# Bans of WHO Class I Pesticides in Bangladesh—suicide prevention without hampering agricultural output

**DOI:** 10.1093/ije/dyx157

**Published:** 2017-08-18

**Authors:** Fazle Rabbi Chowdhury, Gourab Dewan, Vasundhara R Verma, Duleeka W Knipe, Ishrat Tahsin Isha, M Abul Faiz, David J Gunnell, Michael Eddleston

**Affiliations:** 1 Department of Medicine, Sylhet MAG Osmani Medical College, Sylhet, Bangladesh; 2 Centre for Tropical Medicine and Global Health, Nuffield Department of Medicine, University of Oxford, Oxford, UK; 3 OSD, Health Directorate (DGHS), Dhaka, Bangladesh; 4 Department of Medicine, Rangamati Medical College, Rangamati, Bangladesh; 5 Pharmacology, Toxicology, & Therapeutics, University/BHF Centre for Cardiovascular Science, University of Edinburgh, Edinburgh, UK; 6 School of Social and Community Medicine, University of Bristol, Bristol, UK; 7 Dev Care Foundation, Dhaka, Bangladesh; 8 South Asian Clinical Toxicology Research Collaboration, University of Peradeniya, Peradeniya, Sri Lanka

**Keywords:** Bangladesh, pesticides, suicide, prevention, agriculture

## Abstract

**Background:**

Pesticide self-poisoning is a major problem in Bangladesh. Over the past 20-years, the Bangladesh government has introduced pesticide legislation and banned highly hazardous pesticides (HHPs) from agricultural use. We aimed to assess the impacts of pesticide bans on suicide and on agricultural production.

**Methods:**

We obtained data on unnatural deaths from the Statistics Division of Bangladesh Police, and used negative binomial regression to quantify changes in pesticide suicides and unnatural deaths following removal of WHO Class I toxicity HHPs from agriculture in 2000. We assessed contemporaneous trends in other risk factors, pesticide usage and agricultural production in Bangladesh from 1996 to 2014.

**Results:**

Mortality in hospital from pesticide poisoning fell after the 2000 ban: 15.1% vs 9.5%, relative reduction 37.1% [95% confidence interval (CI) 35.4 to 38.8%]. The pesticide poisoning suicide rate fell from 6.3/100 000 in 1996 to 2.2/100 000 in 2014, a 65.1% (52.0 to 76.7%) decline. There was a modest simultaneous increase in hanging suicides [20.0% (8.4 to 36.9%) increase] but the overall incidence of unnatural deaths fell from 14.0/100 000 to 10.5/100 000 [25.0% (18.1 to 33.0%) decline]. There were 35 071 (95% CI 25 959 to 45 666) fewer pesticide suicides in 2001 to 2014 compared with the number predicted based on trends between 1996 to 2000. This reduction in rate of pesticide suicides occurred despite increased pesticide use and no change in admissions for pesticide poisoning, with no apparent influence on agricultural output.

**Conclusions:**

Strengthening pesticide regulation and banning WHO Class I toxicity HHPs in Bangladesh were associated with major reductions in deaths and hospital mortality, without any apparent effect on agricultural output. Our data indicate that removing HHPs from agriculture can rapidly reduce suicides without imposing substantial agricultural costs.

**Key Messages**

- Between 1996 and 2007, 21 pesticides were partially or completely banned by the Bangladeshi regulator, resulting in a shift towards the use of less hazardous WHO toxicity class II, III and U pesticides. All WHO Class I toxicity HHPs were banned in 2000.
- During the post-ban years (2001–14), there were 35 071 (95% CI 25 959 to 45 666) fewer pesticide suicide deaths in Bangladesh compared with pre-ban period (1996–2000).
- There was also evidence of a decline in total unnatural deaths in the post-ban years, with an estimated 76 642 (95% CI 53 493 to 103 161) fewer unnatural deaths.
- The pesticide regulation had an influence on pesticide suicide and overall unnatural death rates without any apparent harmful effect on agricultural production.

## Introduction

Pesticide self-poisoning is a major global means of suicide, responsible for around 150 000 deaths each year.^1–5^ Widespread use of pesticides in rural Asian communities allows easy access in households and through vendors at times of stress.^6^^,^^7^ A key means of reducing the global suicide rate is to reduce access to highly hazardous pesticides (HHPs) through reducing their use in agriculture, safer use and storage, and particularly through regulating and banning HHPs.^4^^,^^8–11^ Additional approaches to suicide prevention include surveillance, means restriction, media guidelines, stigma reduction and raising of public awareness, as well as training for health workers, educators, police and other gatekeepers.^4^

Bangladesh is an agrarian country (current population 161 million in 2015)^12^ with agriculture responsible for 30% of the country's gross domestic product (GDP), 51% of the labour force and >90% of rural employment.^13^ There is extensive use of pesticides in agriculture.^14^ Self-poisoning by pesticide is a serious health problem responsible for about 40% of poisoning cases admitted to hospital and 8–10% of overall mortality in medical wards.^15^^,^^16^ According to government statistics, it is the second most common cause of hospitalization and ninth most common cause of death.^17^ The exact burden of pesticide-related suicide is unknown in Bangladesh. In addition to acute poisoning, pesticides have also been associated with chronic diseases such as cancer, endocrine disruption and neurological disease as well as pollution of the ecosystem.^3^

Synthetic insecticides were introduced in agricultural practice in the 1950s (Supplementary Table 1, available as Supplementary data at *IJE* online).^14^ Over the past two decades, the Bangladesh Government has introduced pesticide legislation, established government bodies to implement the legislation and removed HHPs from agricultural use. The effect of this government action on national suicide rates from pesticide poisoning and from all causes, as well as its effect on agricultural output, has not thus far been investigated. In this study, we found that pesticide regulation was associated with a major reduction in the incidence of suicide by pesticide poisoning and in overall unnatural death rates, without any apparent harmful influence on agricultural production.

## Methods

We collected national Bangladeshi data on pesticide regulatory activities, suicide rates, pesticide poisoning case fatality in hospital, pesticide imports and usage, agricultural outputs and risk factors for suicide, for the years 1996 to 2014.^15^

### National pesticide regulation

Information on national pesticide regulatory activities was obtained from the Plant Protection Wing, Department of Agricultural Extension, Ministry of Agriculture, Government of Bangladesh, and from discussions with people working in the Bangladeshi pesticide industry.

### Suicide data

Suicide data were obtained from the Statistics Division of the Bangladesh Police, which has collected national data on annual unnatural deaths since 1996. These police data identify suicides due to the two main forms, pesticide poisoning (ICD-10: X68) and hanging suicides (X70), while grouping all other forms of suicide in unnatural deaths.^18^ Other categories within the unnatural deaths classification included road traffic crashes, railway and waterway accidents, falls from height, construction injuries, snake bite, drowning, electrocution, lightning injuries and burn-related deaths. No sex- or age-specific data were recorded. Annual population data estimates for 1996 to 2014 were taken from the World Bank.^19^

### Data on risk factors for suicide

Unemployment and agriculture employment data were obtained from the World Bank^19^^,^^20^ and data on divorce rates for 2002 to 2010 from the Bangladesh Bureau of Statistics.^21^ Data on alcohol misuse were obtained from a paper summarizing yearly reports for 2006 to 2011 of the Department of Narcotics Control (DNC), Bangladesh, and from the World Health Organization’s (WHO’s) *Global Status Report on Alcohol and Health* (for 2004, 2011 and 2014).^22^^,^^23^

### Data on crop production and pesticide use

Longitudinal trend data on rice production (the principal national crop) for Bangladesh and other South Asian countries were obtained from the Statistical Division of the Food and Agricultural Organization of the United Nations (FAO) and from the International Rice Research Institute.^24^^,^^25^ Pesticide use data were obtained from the Bangladesh Pesticide Association and from the *Journal of International Development and Cooperation.*^26^ Pesticides were classified according to the WHO Classification of Pesticides (Class Ia (extremely hazardous), Class Ib (highly hazardous), Class II (moderately hazardous), Class III (slightly hazardous), and Class U (unlikely to present acute hazards).^27^

### Data analysis

Data analysis was performed on GraphPad Prism 7 and Stata version 14. We examined trends in overall unnatural deaths, suicides by pesticide poisoning or hanging, and other causes of unnatural deaths, graphically using descriptive statistics. There was statistical evidence of over-dispersion in the Poisson regression models and therefore we used negative binomial regression to quantify changes in the rate and number of: (i) pesticide suicides; and (ii) unnatural deaths, following removal of all Class I pesticides from agricultural use in 2000. We calculated rate ratios (and the change in the number of suicides) for each year in 2001–14 compared with predicted rates based on extrapolated trends before the ban (1996–2000). Negative binomial regression models included a single trend term for calendar year and a dummy variable for each of the post-ban years (14 dummy variables: 2001–14). We carried out sensitivity analyses using 1999 and 2001 as cut-points for pre-/post-ban effects, consistent with the approach used in our recent analysis of the impact of the most recent pesticide legislation in Sri Lanka.^28^

Using the rate ratios and confidence intervals from the primary negative binomial model, we calculated the number of expected pesticide and unnatural deaths for each of the post-ban years (i.e. after 2000). We did this by dividing the observed number of pesticide suicide and unnatural deaths by the rate ratio estimates and confidence intervals. We subtracted the number of expected deaths (pesticide suicide and unnatural) based on pre-ban trends (1996–2000) from the observed number of deaths. In the absence of age-specific mortality data, all analyses were based on crude mortality rates.

## Results

### Pesticide regulation

Between 1996 and 2007, 21 pesticides were partially or completely banned by the Bangladeshi regulator (Table 1), resulting in a shift towards the use of less hazardous WHO toxicity classes II, III and U pesticides. Organochlorine compounds were the main pesticides used in Bangladesh from 1950 until their withdrawal in the mid 1990s.^14^ The widely used organophosphorus (OP) insecticide HHPs were banned at the end of 2000 when all Class I pesticides were banned from agricultural practice.^14^

**Table 1.** Pesticides banned or withdrawn from agricultural practice in Bangladesh

Year	Compound(s)	Reasons for ban/withdrawal
1960	Endrin^w^	Toxic to fish and aquatic organisms
1997	Chlordane,^w^ DDT,^a^ dieldrin,^b^ heptachlor^w^	Phasing out of persistent organic pollutants (POPs)
1998	Pyrethroids^b^	Toxic to fish and aquatic organisms
	Endosulfan^b^	Environmental concerns
2000	Dichlorvos,^b^ dicrotophos,^b^ disulfoton,^b^ ethyl parathion,^b^ methyl parathion,^b^ mercury compounds,^b^ monocrotophos ^b^ phosphamidon^b^	Removal of all class Ia and Ib pesticides from agricultural use
2004	Methyl bromide^b^	Montreal protocol on Ozone Layer Depleting Substances (1987)
2007	Hexachlorobenzene,^b^ mirex,^b^ toxaphene^b^	Stockholm Treaty on Persistent Organic Pollutants (2001)

^a^Restricted use only permitted in vector control.

^b^Banned for use on rice and other lowland crops.

^W^Withdrawn for all uses.

### Correlation between regulations on pesticides and case fatalities in hospitals

To reflect the active implementation of pesticide legislation in late 1990s, we compared data for indicators of pesticide poisoning up to and after 2000 (date of the key ban of the most toxic WHO Class I toxicity HHPs, Table 2) using data from the literature.^15^ There was a 37.1% [95% confidence interval (CI) 35.4 to 38.8%] relative reduction in case fatality from pesticide poisoning, falling from 413/2719 (15.1%, 95% CI 13.9 to 16.6%) in the years up to 2000 to 315/3296 (9.5%, 95% CI 8.6 to 10.6%) after 2001 (*P* < 0.0001). No difference in rate of hospital admission for pesticide poisoning or the proportion of self-poisoning cases using pesticides was noted (Table 2).

**Table 2.** Comparison of common pesticide poisoning indicators pre- and post-bans of HHPs^15^

Indicators	Time frame		*P*-value
	1970 to 2000	2001 to 2014	
Poisoning cases as a proportion of total admissions (%)	3.4 (*n* = 1737)	7.1 (*n* = 6456)	<0.001
Pesticide poisoning admission rate (per 100 000/year)	2.9^a^	3.1	0.57
Proportion of self-poisoning using pesticides (%)	70.1 (*n* = 240)	72.2 (*n* = 5316)	0.40
In-hospital case fatality from pesticide poisoning (%)	15.1 (*n* = 413)	9.5 (*n* = 315)	<0.001

^a^Data for the period 1988 to 2000.

### Influence of pesticide regulation on pesticide suicides

From 1996 to 2014, the Bangladesh Police Statistics Department recorded 311 208 unnatural deaths. Intentional pesticide self-poisoning was the most common cause of suicidal death, being responsible for 115 423 [37.1%, 95% confidence interval (CI) 36.9% to 37.3%] of these deaths. Hanging and other causes of unnatural death were responsible for 95 063 (30.5%, 95% CI 30.4 to 30.7%) and for 100 722 (32.4%, 95% CI 32.2 to 32.5%) deaths, respectively. These proportions changed over time: in 1996, 44.7% of unnatural deaths were due to pesticide suicides compared with 20.9% in 2014. Hanging and other causes of unnatural deaths increased from 25.2% and 30.1% in 1996 to 40.1% and 38.9% in 2014, respectively.

**Figure 1:**
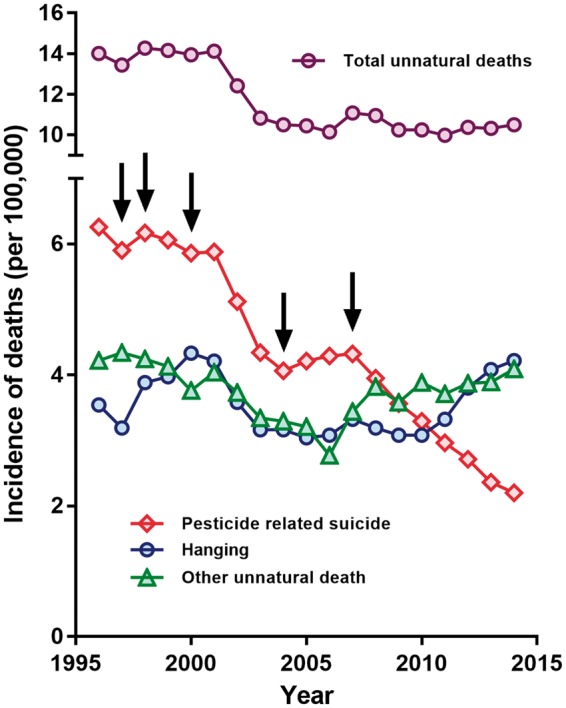
Trends of unnatural deaths in Bangladesh 1996 to 2014. The arrows mark the timing of national pesticide regulation, see Table 1 for timing of the bans. Suicides not using either pesticides or hanging are included in the ‘Other unnatural death’ category.

Before 2000, the rates of all causes of unnatural death were fairly constant, except for hanging suicides which appear to have increased over this time (Figure 1). From 2002 onwards, there was a sharp decline in the pesticide suicide death rate, as well as a decline in hanging suicides and other causes of unnatural death. The decline in pesticide suicides continued until 2005, at which point there was a slight rise followed by a decline from 2008 onwards. The fall in hanging suicides continued from 2000 until 2003 when the rate plateaued before steadily rising from 2011 onwards (Figure 1). The mortality rate due to other causes of unnatural deaths fell between 2000 and 2006 before increasing from 2007 onwards.

The incidence of pesticide suicides declined by 65.1% (95% CI 52.0 to 76.7%) over this period, from 6.3/100 000 in 1996 to 2.2/100 000 in 2014 (Figure 1). At the same time, there was a 20.0% (95% CI 8.4 to 36.9%) increase in the incidence of hanging from 3.5/100 000 in 1996 to 4.2/100 000 in 2014, and a 2.4% (95% CI 0.1 to 12.6%) decrease in other causes of unnatural death from 4.2/100 000 in 1996 to 4.1/100 000 in 2014. The incidence of all unnatural deaths fell by 25.0% (95% CI 18.1 to 33.0%), from 14.0/100 000 in 1996 to 10.5/100 000 in 2014.

**Figure 2:**
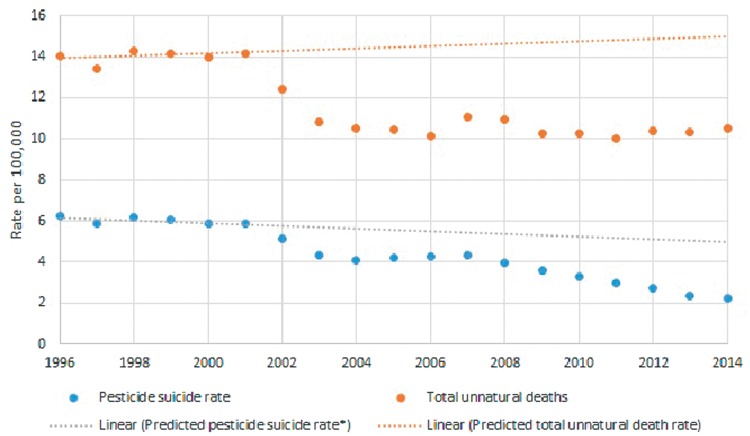
Rates of pesticide suicides and unnatural deaths in 1996–2014, with predicted line based on trend in 1996 2000.

The pesticide suicide rate was lower in each post-ban year (2001–14) than would be expected based on previous trends (1996 2000) (Table 3 and Figure 2). We estimate that in the post-ban years there were 35 071 (95% CI 25 959 to 45 666) fewer pesticide suicide deaths than predicted based on trends between 1996 and 2000. There was also evidence of a decline in total unnatural deaths in the post-ban years, with an estimated 76 642 (95% CI 53 493 to 103 161) fewer unnatural deaths. Our sensitivity analyses, which altered the start of the post-ban period from 2000 to 1999 and 2001, showed results consistent with our primary analysis (Table 4).

**Table 3.** Rate ratios and change in the number of pesticide suicides and total unnatural deaths in years 2001–14, after ban of WHO class I pesticides, relative to those expected based on pre-band trend 1996–2000

	Pesticide suicides			Unnatural deaths		
	Rate ratio (95% CI)	*P-*values	Change in number of suicides (95% CI)	Rate ratio (95% CI)	*P*-values	Change in number of deaths (95% CI)
Post ban years^a^						
2001	1.00 (0.98, 1.03)	0.79	28 (−179, 229)	1.00 (0.98, 1.02)	0.93	−21 (−471, 419)
2002	0.88 (0.85, 0.91)	<0.001	−919 (−1198, −649)	0.87 (0.85, 0.90)	<0.001	−2421 (−3039, −1823)
2003	0.76 (0.72, 0.79)	<0.001	−1938 (−2297, −1595)	0.76 (0.73, 0.79)	<0.001	−4764 (−5571, −3988)
2004	0.71 (0.68, 0.75)	<0.001	−2277 (−2719, −1858)	0.73 (0.70, 0.77)	<0.001	−5379 (−6391, −4416)
2005	0.75 (0.70, 0.80)	<0.001	−2008 (−2536, −1513)	0.73 (0.69, 0.77)	<0.001	−5624 (−6852, −4465)
2006	0.77 (0.72, 0.83)	<0.001	−1830 (−2444, −1258)	0.70 (0.66, 0.75)	<0.001	−6236 (−7690, −4876)
2007	0.79 (0.72, 0.86)	<0.001	−1714 (−2417, −1067)	0.76 (0.71, 0.82)	<0.001	−5025 (−6715, −3460)
2008	0.73 (0.66, 0.80)	<0.001	−2196 (−2988, −1475)	0.75 (0.69, 0.82)	<0.001	−5358 (−7291, −3583)
2009	0.66 (0.60, 0.73)	<0.001	−2721 (−3603, −1926)	0.70 (0.64, 0.77)	<0.001	−6624 (−8811, −4634)
2010	0.62 (0.55, 0.69)	<0.001	−3078 (−4052, −2209)	0.70 (0.63, 0.77)	<0.001	−6738 (−9191, −4529)
2011	0.56 (0.50, 0.64)	<0.001	−3526 (−4593, −2583)	0.68 (0.60, 0.76)	<0.001	−7320 (−10048, −4885)
2012	0.52 (0.45, 0.60)	<0.001	−3877 (−5039, −2861)	0.70 (0.62, 0.79)	<0.001	−6899 (−9915, −4233)
2013	0.46 (0.40, 0.53)	<0.001	−4382 (−5640, −3293)	0.69 (0.61, 0.79)	<0.001	−7162 (−10478, −4258)
2014	0.43 (0.37, 0.50)	<0.001	−4605 (−5961, −3443)	0.70 (0.61, 0.81)	<0.001	−7071 (−10698, −3924)

^a^Compared with pre-ban trend 1996–2000.

**Table 4.** Sensitivity analysis changing the post-ban period from 2001–14 to 2000–14 and 2002–14

	Pesticide suicides rate ratio (95% CI)		Unnatural deaths rate ratio (95% CI)	
	Start of ban		Start of ban	
	1999	2001	1999	2001
Post ban years				
2000	0.97 (0.95, 1.00)		0.98 (0.95, 1.00)	
2001	0.98 (0.95, 1.02)		0.98 (0.95, 1.01)	
2002	0.86 (0.82, 0.90)	0.88 (0.87, 0.90)	0.85 (0.81, 0.89)	0.88 (0.86, 0.89)
2003	0.73 (0.69, 0.78)	0.75 (0.73, 0.77)	0.74 (0.70, 0.78)	0.76 (0.74, 0.78)
2004	0.69 (0.64, 0.74)	0.71 (0.69, 0.74)	0.71 (0.66, 0.76)	0.73 (0.71, 0.76)
2005	0.72 (0.66, 0.79)	0.75 (0.72, 0.78)	0.70 (0.64, 0.76)	0.73 (0.70, 0.75)
2006	0.74 (0.66, 0.82)	0.77 (0.73, 0.81)	0.67 (0.61, 0.74)	0.70 (0.67, 0.73)
2007	0.75 (0.66, 0.84)	0.78 (0.74, 0.83)	0.73 (0.65, 0.81)	0.77 (0.73, 0.80)
2008	0.69 (0.60, 0.78)	0.72 (0.68, 0.77)	0.71 (0.63, 0.80)	0.75 (0.71, 0.80)
2009	0.62 (0.54, 0.72)	0.66 (0.61, 0.71)	0.66 (0.58, 0.75)	0.70 (0.66, 0.75)
2010	0.58 (0.49, 0.68)	0.61 (0.57, 0.66)	0.65 (0.57, 0.76)	0.70 (0.65, 0.75)
2011	0.52 (0.44, 0.62)	0.56 (0.51, 0.61)	0.63 (0.54, 0.74)	0.68 (0.63, 0.73)
2012	0.48 (0.40, 0.58)	0.52 (0.47, 0.57)	0.65 (0.55, 0.77)	0.70 (0.65, 0.76)
2013	0.42 (0.35, 0.51)	0.45 (0.41, 0.50)	0.64 (0.53, 0.77)	0.70 (0.63, 0.76)
2014	0.39 (0.32, 0.49)	0.43 (0.38, 0.48)	0.65 (0.53, 0.79)	0.70 (0.64, 0.78)

### Influence of pesticide regulation on rice production

**Figure 3:**
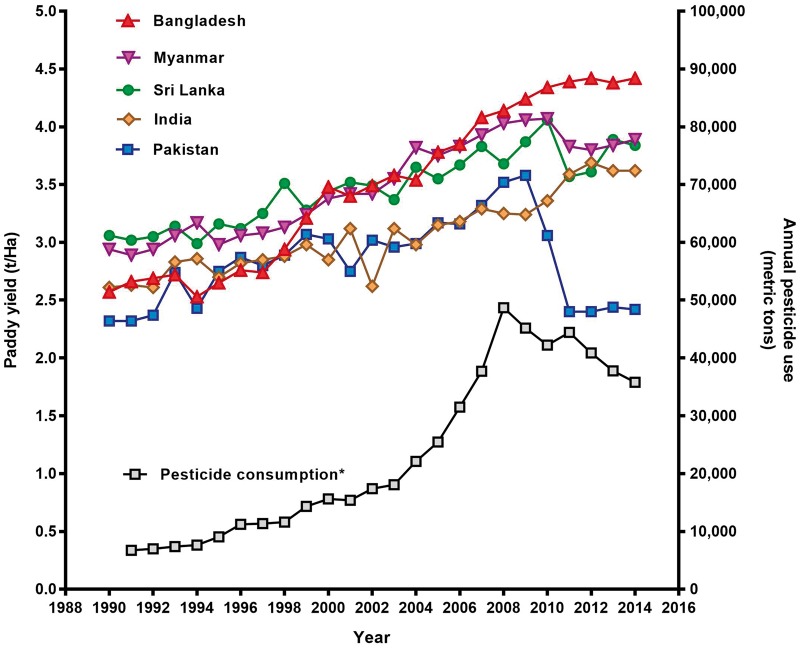
Pesticide use and rice production 1990 to 2014.^2^^4^^,^^26^^,^^51^

The main crop of Bangladesh is rice paddy, accounting for 75% of total agricultural land use and 80% of pesticide use.^14^ Comparing annual rice production in Bangladesh with production in India, Pakistan, Sri Lanka and Myanmar from 1996 to 2014 (Figure 3) revealed no apparent influence of the pesticide bans on crop production. Of note, overall pesticide, and insecticide, consumption increased for much of the period during which there was the rapid reduction in incidence of pesticide suicides.

### Risk factors for suicide

Although data were not complete for the whole period, we could not find evidence that the reduction in pesticide suicides was brought about by changes in employment, divorce or alcohol use rates. Unemployment levels varied between a minimum of 2.5% and a maximum of 5.0% of the total labour force between 1991 and 2014; levels were 2.5% in 1996 and 4.3% in 2014.^19^ The size of the work force in the agricultural sector also did not vary markedly during the study period. According to the World Bank, from 1984 to 2010, the percentage of total direct employment in the agricultural sector ranged from 48% to 66%.^20^ Since 2000, about 87% of rural households rely on agriculture for at least part of their income.^29^ The absolute number of the work force (aged 15+) was 36.0 million in 1995–96, increasing to 56.7 million in 2010.^20^

Available data suggest that the divorce rate increased only modestly in Bangladesh during this period: increasing among women and men from 0.95 to 1.22/1000 and 0.29 to 0.40/1000, between 2002 and 2010, respectively.^21^ Alcohol consumption is also increasing in Bangladesh but is still low compared with global use, with an estimated 1.9% (95% CI 1.7 to 2.1) of the population using alcohol [men: 3.6% (95% CI 3.3 to 4.1), women 0.3% (95% CI 0.2–0.5)] in 2010.^22^^,^^23^ There are no data to suggest a significant change in alcohol misuse patterns at the same time as the marked fall in pesticide suicides in the 2000s; unfortunately, national data from before 2004 were not available.

## Discussion

In this study, we assessed the association of pesticide regulations carried out in Bangladesh, in particular the ban of all WHO Class I toxicity HHPs in 2000, with changes in pesticide suicides and agriculture. We found that, although use of pesticides for self-poisoning remained steady over the past two decades, there were major 37.1%, 65.1% and 25.0% reductions in the case fatality for pesticide poisoning and incidence of pesticide suicides and total unnatural deaths, respectively. At the same time, we found no apparent influence on the production of rice paddy, the country’s key agricultural crop and food staple, as well as increased pesticide use. We found no indication that changes in known contributors to suicide rates—unemployment, alcohol misuse or divorce^30^—had changed significantly during this period.

Legislative removal of the most hazardous pesticides from agricultural practice by the Bangladeshi Government is the most likely reason for these effects, as has been noted previously in Sri Lanka^31^^,^^32^ and South Korea.^33^ In the 14 years following the pesticide regulations in 2000, there were 35 000 fewer suicides by pesticide poisoning than expected based on trends between 1996 and 2000; this is a similar number to the tens of thousands of suicides that pesticide regulation prevented in Sri Lanka after 1995.^34^ The chronological association between pesticide legislation and reductions in pesticide suicide are not so clear in Bangladesh as they were in Sri Lanka, but the trend is clear. The difference is likely due to the comparative ease of controlling pesticide imports and use in an island like Sri Lanka compared with Bangladesh with its multiple land borders across which imports may cross.

The lack of an association with changes in paddy yield is important, since a detrimental effect on agricultural costs and yields is the major concern with pesticide legislation for HHPs. The data from Bangladesh add to the supportive data from both Sri Lanka^35^ and South Korea,^33^ indicating that careful pesticide legislation can reduce suicides without clearly affecting agricultural output.

China and Taiwan have also seen marked reductions in suicide rates from pesticide poisoning;^36–38^ however, these reductions may have been more associated with urbanization and reduction in the agricultural work force than pesticide regulation.^36^^,^^39^^,^^40^

Hospital-based studies have shown that, from 2005 onwards, the majority of poisoning admissions in Bangladesh have been due to a mixture of Class II and Class III products rather than the previously common WHO class I pesticides.^15^ At present, the WHO class III insecticide malathion is the commonest agent used for self-poisoning.^15^ The in-hospital mortality from pesticide poisoning in Bangladesh after the legislation was found to be 37% lower than before the bans. We hypothesize that this is due to the resulting reduced toxicity of pesticides taken in self-harm; however, bans of highly hazardous pesticides with switches to moderately but still toxic pesticides can actually result in more patients dying in hospital, since the former often resulted in people dying quickly, before presentation to health services.^41^ It is also possible that the reduced case fatality was due to increased awareness among physicians of how to treat these patients, increased local research on pesticide poisoning management,^42^^,^^43^ the publication and use of national guidelines for poisoning management in 2005^44^ and/or better management of admitted patients. However, there is no evidence of a consistent marked improvement in the management of pesticide-poisoned patients during this period. The lack of adequate training for physicians, a shortage of antidotes and a lack of intensive care unit (ICU) facilities means that moderate-to-severe pesticide poisoning remains a challenging issue in Bangladesh.^15^

National representative data on the annual incidence of suicides in Bangladesh are not yet available. We therefore used police data collected from whole country only for this report. In 2001, the WHO estimated the national suicide rate to have been 8/100 000 in 1972–88 and 10/100 000 in 1992–93.^45^ More recently, the WHO estimated the national suicide rate to be 6.6/100 000 in 2012.^4^ A cross-sectional study in 2003 of a population of 819 429 found a suicide rate of 7.3 (95% CI 5.6 to 9.5) per 100 000 per year.^46^ These estimates are similar to the estimated incidence in police data presented here of 10.4/100 000 for all unnatural deaths, including at least 6.51/100 000 suicides (hanging + pesticide poisoning). It is surprising that non-suicide injury deaths in Bangladesh are estimated to be as low as 4/100 000 per year. It is possible that these injuries are not all reported to the police.

Suicide rates are likely to be higher in rural areas.^47^^,^^48^ Studies in rural Jessore district^49^ and Matlab upazila (sub-district) of Chandpur district^50^ found rates of 39/100 000 during 1983–2002, and 13/100 000 in women and 8/100 000 per year in men in 1982–98, respectively. The large cross-sectional study reported above found suicide rates to be 17-fold higher (95% CI 5.4 to 54.6) in the rural population, compared with urban rates.^46^ This is likely to be due to the easy availability of pesticides in these rural communities, as well other characteristics of rural life.

### Limitations

Our study is based on data from the police statistics division, which have not been validated against other sources of information on suicides. Since suicide is a crime in Bangladesh, it is likely to be under-reported, lowering the absolute rates but probably not affecting the rates over time. Absence of age- and sex-specific stratified suicide records was a limitation of the analysed data. Data for many of the risk factors were only available for some of the period under study, and so it was not possible to completely rule out the role of other risk factors.

### Conclusions

Removal of HHPs from agricultural practice by government legislation was associated with a marked reduction in suicide by pesticide poisoning, without affecting agricultural outputs. Widespread global adoption of this practice will rapidly reduce global suicide numbers, by preventing nearly all pesticide suicides. The data presented here add to data already collected from Sri Lanka and South Korea.

## Supplementary Data

Supplementary data are available at *IJE* online.

## Funding

DK is an Economic and Social Research Council (U.K) postdoctoral fellow (ES/P009735/1).

## Supplementary Material

Supplementary Figure

Supplementary Web Table 1
