# Supplementary figures and images for: Bans of WHO Class I Pesticides in Bangladesh—suicide prevention without hampering agricultural output
Source: Int J Epidemiol. 2017 Aug 18;47(1):175–84. doi: 10.1093/ije/dyx157 (PMC5837375; doi:10.1093/ije/dyx157)

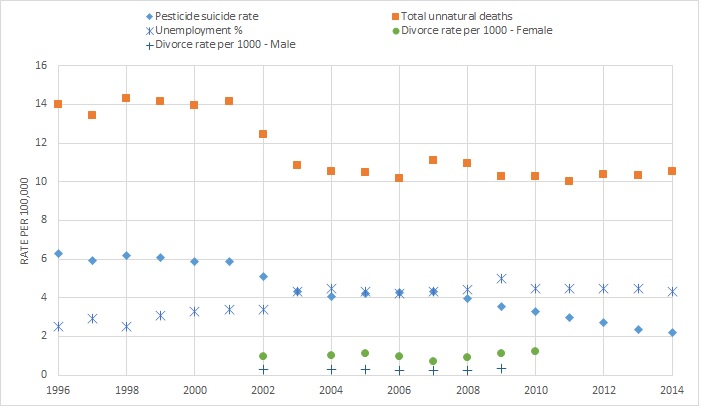

Supplement: Supplementary Figure [file dyx157_ije-2016-12-1472-file006.jpeg]
