# Supplementary material for: Bans of WHO Class I Pesticides in Bangladesh—suicide prevention without hampering agricultural output
Source: Int J Epidemiol. 2017 Aug 18;47(1):175–84. doi: 10.1093/ije/dyx157 (PMC5837375; doi:10.1093/ije/dyx157)
Supplement: Supplementary Web Table 1 [file dyx157_ije-2016-12-1472-file007.docx]

**Web Table 1**

**Historical background of introduction of pesticides and regulation into Bangladesh (14, 52, 53)**

| **Year** | **Events** |
| --- | --- |
| 1950s | Pesticide use starts with introduction of endrin into agricultural practice |
| 1960s | Distribution of pesticides and spraying equipment free of cost to farmers (until 1975 when it was partially subsidized until 1979) |
| 1960s | The organochlorines endrin, aldrin and lindane are banned due to environmental concerns |
| 1971 | The Pesticide Ordinance, 1971, comes into force, providing legislation to regulate the import, manufacture, formulation, sale, distribution and use of pesticides in Bangladesh |
| 1979 | Government withdraws all subsidies from pesticides. Importation and sales of pesticides are handed over to the private sector |
| 1985 | Enactment of Pesticide Rules legislation to regulate pesticide registration, formulation, packaging and sales in Bangladesh, and the function of the advisory committees and state laboratory |
| 1986 | Registration of pesticide after review by the national Pesticide Technical Advisory Committee (PTAC) becomes mandatory |
| 1992 | A National Environment Policy is formulated which initiates the process of phasing out and banning persistent organic pollutants (POPs), including organochlorine insecticides |
| 2002 | Integrated pest management policy adopted nationally |
